# Metformin Attenuates Sepsis-Induced Cardiomyopathy via Inhibition of Reverse Electron Transfer at Mitochondrial Complex I

**DOI:** 10.3390/biology15151267

**Published:** 2026-08-02

**Authors:** Nannan He, Wen Cao, Yannian Luo, Chao Sun, Ting Zhao, Xiongxiong Liu, Meiling Li, Lin Wei, Bing Wang, Jian Liu

**Affiliations:** 1 The First School of Clinical Medicine, Lanzhou University, Lanzhou 730000, China; ldyy_henn@lzu.edu.cn; 2 Department of Critical Care Medicine, Lanzhou University Second Hospital, Lanzhou 730000, China; smilecao2009@126.com (W.C.); 18215103861@163.com (M.L.); 3 Department of Critical Care Medicine, Gansu Province Hospital of TCM, Lanzhou 730000, China; luoyn405@163.com; 4 Institute of Modern Physics, Chinese Academy of Sciences, Lanzhou 730000, China; sunchao@impcas.ac.cn (C.S.); zhaoting@impcas.ac.cn (T.Z.); lxx002@impcas.ac.cn (X.L.); 5 State Key Laboratory of Heavy Ion Science and Technology, Lanzhou 730000, China; 6 Gansu Provincial Maternity and Child-Care Hospital/Gansu Provincial Center Hospital, Lanzhou 730000, China; weil_gsfy1@sina.com; 7 Institute for Radiological Science, National Institutes for Quantum Science and Technology (QST), Chiba 263-8555, Japan

**Keywords:** sepsis-induced cardiomyopathy, reverse electron transfer, mitochondrial ROS, complex I, metformin

## Abstract

Sepsis-induced cardiomyopathy (SICM) is a life-threatening complication of sepsis; however, its molecular mechanisms remain incompletely understood, which has hindered the development of targeted therapies. We hypothesized that excessive mitochondrial reactive oxygen species (mtROS) production through reverse electron transfer (RET) at mitochondrial complex I contributes to septic myocardial injury and that metformin, a clinically used inhibitor of mitochondrial complex I, protects the myocardium by inhibiting this process. In lipopolysaccharide-stimulated H9C2 cardiomyocytes and cecal ligation and puncture-induced septic rats, sepsis was characterized by an elevated mitochondrial membrane potential, accompanied by succinate accumulation, an increased NADH/NAD^+^ ratio, and impaired downstream electron transport. These metabolic changes established favorable conditions for RET-mediated mtROS generation. Metformin inhibited complex I activity and selectively suppressed RET-mediated mtROS generation without increasing ROS production associated with forward electron transport (FET). This effect was accompanied by attenuated inflammatory responses and apoptosis. In septic rats, metformin preserved cardiac function and alleviated myocardial oxidative stress and injury. Overall, these results suggest that RET at mitochondrial complex I represents a potential therapeutic target in SICM and support the use of metformin as a promising strategy for preventing and treating septic myocardial dysfunction.

## Simple Summary

Sepsis can lead to severe and life-threatening cardiac damage, referred to as sepsis-induced cardiomyopathy. At present, clinical management of this condition is mainly supportive, and effective targeted treatments to protect cardiac function are still lacking. In this study, we explored the mechanisms contributing to sepsis-associated myocardial injury and investigated the cardioprotective effects of metformin, a widely prescribed medication with a well-established safety profile. Our findings demonstrated that sepsis impaired mitochondrial function and triggered excessive mitochondrial reactive oxygen species generation, which further promoted inflammatory responses, apoptosis, and cardiac dysfunction. Metformin effectively suppressed these pathological changes by maintaining mitochondrial energy metabolism. In cardiomyocyte and septic rat models, metformin attenuated inflammatory responses and cell death, alleviated myocardial injury, and preserved cardiac function. Considering its extensive clinical use, favorable safety profile, and widespread availability, metformin may offer a promising strategy for rapid clinical translation to protect cardiac function in patients with sepsis and help address this important unmet medical challenge.

## 1. Introduction

Sepsis-induced cardiomyopathy (SICM) is a severe complication of sepsis, clinically characterized by reversible myocardial depression, including isolated or biventricular systolic and/or diastolic dysfunction [1]. Recent multicenter studies have reported a highly variable prevalence of SICM, ranging from 5% to 79% in patients with severe sepsis and septic shock. This variation may be attributed to differences in diagnostic criteria, patient characteristics, and the timing of cardiac evaluation [2]. The occurrence of SICM is associated with a 2–3-fold increase in 28-day all-cause mortality, and mortality can exceed 70% in patients with refractory cardiac dysfunction complicated by multiorgan failure [3]. Despite growing recognition of SICM clinical phenotype and prognostic impact, the molecular mechanisms driving myocardial injury during SICM remain incompletely understood. Current treatment relies primarily on supportive interventions, including fluid resuscitation, vasopressors, and inotropic agents, which fail to directly target intrinsic myocardial injury. Therefore, identifying effective mechanism-oriented treatments for SICM remains an important clinical priority [4,5]. 

Mitochondrial dysfunction has been implicated as a key pathological event in sepsis-associated organ dysfunction [6]. Excessive mitochondrial reactive oxygen species (mtROS) production represents an important mechanism linking disturbed mitochondrial metabolism with myocardial damage, inflammatory activation, and apoptosis [7]. Although mtROS can originate from multiple mitochondrial sites, reverse electron transfer (RET) through complex I has attracted increasing attention as a potent source of oxidative stress in conditions such as ischemia–reperfusion injury, chronic inflammation, and cancer progression [8,9,10]. RET occurs when an elevated proton motive force and an over-reduced coenzyme Q (CoQ) pool drive electrons in the reverse direction from ubiquinol toward complex I, generating superoxide at the flavin mononucleotide site [11,12]. This reverse electron flow is favored by a specific metabolic state characterized by succinate accumulation, an elevated NADH/NAD^+^ ratio, and impaired electron transfer through downstream respiratory complexes [8,13]. In sepsis, succinate accumulation has been consistently observed in experimental models and clinical samples, and elevated circulating succinate levels correlate with increased inflammatory cytokine levels and worse clinical outcomes [14,15]. Modulating succinate dehydrogenase (SDH) activity to limit succinate accumulation may shift mitochondrial metabolism away from excessive mtROS generation and promote an anti-inflammatory phenotype [16]. However, whether these sepsis-associated metabolic disturbances are sufficient to induce RET-dependent mtROS generation in the septic myocardium remains unclear. Defining this mechanism may reveal a novel therapeutic target for SICM.

Metformin is a biguanide widely used as a first-line treatment for type 2 diabetes mellitus and is also recognized as a mild, clinically tolerated inhibitor of mitochondrial complex I [17,18]. In contrast to more hydrophobic biguanides such as phenformin, which was withdrawn because of its association with an increased risk of lactic acidosis, metformin shows a safer therapeutic profile, partly due to its distinct mode of complex I inhibition [19,20]. Recent structural studies have shown that metformin accesses complex I only in its open deactive conformation and becomes trapped at the ubiquinone redox site upon substrate-induced closure [21]. In this configuration, metformin physically blocks electron reflux from the reduced CoQ pool while largely preserving forward respiratory activity [21]. This site-specific action enables metformin to inhibit RET-derived mtROS production without suppressing forward electron transfer (FET). Beyond its glucose-lowering effects, metformin has been associated with cardiovascular benefits in both diabetic and non-diabetic populations [22,23]. Its extensive clinical experience, favorable safety characteristics, and wide availability make it an attractive candidate for repurposing in critically ill conditions [24]. Nevertheless, whether metformin protects against SICM through RET inhibition has not been investigated.

We proposed that sepsis promotes a metabolic state favorable for RET activation in the myocardium and that metformin may protect against septic cardiac injury by limiting RET at mitochondrial complex I. To examine this hypothesis, we established an in vitro model using lipopolysaccharide (LPS)-stimulated H9C2 cardiomyocytes and an in vivo model using cecal ligation and puncture (CLP)-induced septic rats. Through targeted metabolomic profiling, mitochondrial functional analyses, pharmacological interventions, and cardiac functional assessments, we investigated whether metformin selectively inhibits RET-driven mtROS production, attenuates myocardial inflammation and apoptosis, and preserves cardiac function during sepsis. Our findings identify RET at mitochondrial complex I as a therapeutically targetable mechanism in SICM and support metformin as a potential therapeutic strategy for septic myocardial dysfunction.

## 2. Materials and Methods

### 2.1. Key Reagents and Resources

LPS (Sigma-Aldrich, St. Louis, MO, USA, L2360; 20 μg/mL in vitro); metformin hydrochloride (MET) (Sigma-Aldrich, St. Louis, MO, USA, D150959; 1 mM in vitro, 100 mg/kg in vivo); MitoQ (MedChemExpress, Monmouth Junction, NJ, USA, HY-100116A; 1 μM in vitro); rotenone (Sigma-Aldrich, St. Louis, MO, USA, R8875; 50 nM in vitro); S1QEL1.1 (S1) (MedChemExpress, Monmouth Junction, NJ, USA; HY-129115; 1 μM in vitro); 12% sodium dodecyl sulfate-polyacrylamide gel electrophoresis (SDS-PAGE) precast gels (Solarbio, Beijing, China, P1200); bicinchoninic acid (BCA) protein assay reagent (Beyotime, Shanghai, China, P0012S); Dulbecco’s modified Eagle medium (DMEM) (MeilunBio^®^, Dalian, China); fetal bovine serum (FBS) (Procell, Wuhan, China); penicillin–streptomycin solution (100×) (Minhai Bio, Lanzhou, China); polyvinylidene difluoride (PVDF) membranes (Servicebio, Wuhan, China, G6015-0.45); radioimmunoprecipitation assay (RIPA) lysis buffer (Solarbio, Beijing, China, R0010); TRIzol reagent (Invitrogen, Carlsbad, CA, USA, 15596026); and H9C2 rat cardiomyocyte cell line (Cell Bank of the Chinese Academy of Sciences, Shanghai, China).

### 2.2. Cell Culture and Treatment

H9C2 cells were cultured in DMEM supplemented with 10% FBS and 1% penicillin–streptomycin at 37 °C in a humidified incubator containing 5% CO_2_. To establish an in vitro model of SICM, cells were stimulated with 20 μg/mL LPS. For intervention studies, cells were treated with metformin (1 mM), S1QEL1.1 (1 μM), rotenone (50 nM), or MitoQ (1 μM), either alone or starting 1 h after LPS stimulation, and maintained for 2, 24, or 48 h according to the requirements of each assay. Untreated cells were used as controls.

### 2.3. Enzyme-Linked Immunosorbent Assay (ELISA)

The concentrations of IL-6, IL-1β, TNF-α, and cTnI in culture supernatants and serum samples were measured using ELISA kits: IL-6 (JL20896, Jianglai Bio, Shanghai, China), IL-1β (JL18365, Jianglai Bio, Shanghai, China), TNF-α (JL13202, Jianglai Bio, Shanghai, China), and cTnI (SEKR-0048, Solarbio, Beijing, China). All procedures followed the manufacturers’ protocols.

### 2.4. Quantitative Real-Time PCR (qRT-PCR)

Total RNA was extracted from H9C2 cells and rat myocardial tissues using TRIzol reagent (Invitrogen, Carlsbad, CA, USA). RNA concentration and purity were determined using a NanoDrop spectrophotometer (Thermo Fisher Scientific, Waltham, MA, USA). Complementary DNA was synthesized using a reverse transcription kit following the manufacturer’s protocol. Quantitative real-time PCR was conducted using GoTaq^®^ qPCR Master Mix (Promega, Madison, WI, USA) on a QuantStudio 5 Real-Time PCR System. *GAPDH* was used as the internal control. The primer sequences used for amplification were as follows: *IL-6* forward 5′-ACTTCCAGCCAGTTGCCTTCTTG-3′, reverse 5′-TGGTCTGTTGTGGGTGGTATCCTC-3′; *IL-1β* forward 5′-AATCTCACAGCAGCATCTCGACAAG-3′, reverse 5′-TCCACGGGCAAGACATAGGTAGC-3′; *TNF-α* forward 5′-CACCACGCTCTTCTGTCTACTGAAC-3′, reverse 5′-TGGGCTACGGGCTTGTCACTC-3′; *cTnI* forward 5′-CCACCGAGCCACATGCCAAG-3′, reverse 5′-TCCTCTGCCTCACGCTCCATC-3′; *GAPDH* forward 5′-AAGTTCAACGGCACAGTCAAGG-3′, reverse 5′-GACATACTCAGCACCAGCATCAC-3′. Relative gene expression levels were calculated using the 2^−ΔΔCT^ method. 

### 2.5. Reactive Oxygen Species (ROS) Detection

Intracellular ROS levels were measured using a 2′,7′-dichlorodihydrofluorescein diacetate (DCFH-DA) assay kit (S0033S, Beyotime, Shanghai, China). Briefly, H9C2 cells were seeded in 6-well plates and subjected to the indicated treatments. Cells were then harvested and incubated with 10 μM DCFH-DA in the dark at 37 °C for 20 min. After three washes with phosphate-buffered saline (PBS), cells were resuspended and immediately analyzed by flow cytometry. Mean fluorescence intensity was quantified using FlowJo software (v10.8.1).

### 2.6. Mitochondrial Superoxide (MitoSOX) Staining

MitoSOX production was detected using MitoSOX Red (M36008, Invitrogen Carlsbad, CA, USA). H9C2 cells cultured on confocal dishes were treated as indicated and incubated with 5 μM MitoSOX Red in PBS for 30 min at 37 °C in the dark. Fluorescence images were immediately acquired using a laser scanning confocal microscope (LSM710, Carl Zeiss, Oberkochen, Germany) with excitation at 510 nm and emission at 580 nm.

### 2.7. Mitochondrial Membrane Potential (ΔΨm) Detection

H9C2 cells were seeded in 35 mm culture dishes and treated under the indicated conditions. After treatment, cells were harvested, resuspended in culture medium, and incubated with an equal volume of JC-10 working solution (CA1310, Solarbio, Beijing, China) for 30 min at 37 °C in the dark. Cells were then washed with staining buffer, resuspended in PBS, and analyzed by flow cytometry.

### 2.8. Mitochondrial Respiratory Chain Complex Activities

The enzymatic activities of mitochondrial respiratory chain complexes I–V were measured in H9C2 cells and rat myocardial tissues using commercial assay kits (Solarbio, Beijing, China) according to the manufacturer’s instructions. The following kits were used: complex I (NADH-CoQ reductase, BC0515), complex II (succinate-CoQ reductase, BC3235), complex III (CoQ-cytochrome c reductase, BC3245), complex IV (cytochrome c oxidase, BC0945), and complex V (ATP synthase, BC1445). Enzyme activities were normalized to cell number (U/10^6^ cells) or tissue weight (U/g tissue).

### 2.9. Western Blot Analysis

Cells and myocardial tissues were lysed in RIPA lysis buffer supplemented with protease and phosphatase inhibitors. Equal amounts of protein were separated by 12% SDS-PAGE and transferred onto PVDF membranes. After blocking with 5% non-fat milk in Tris-Buffered Saline with Tween 20 for 2 h, membranes were incubated with primary antibodies overnight at 4 °C, followed by incubation with horseradish peroxidase (HRP)-conjugated secondary antibodies for 2 h at room temperature. Protein bands were visualized using enhanced chemiluminescence (1705061, Bio-Rad, Hercules, CA, USA) and quantified with ImageJ software (v1.54g). Primary antibodies used were as follows: NDUFA9 (A24H16, Selleck, Houston, TX, USA; 1:1000), SDHC (E11N4, Selleck, Houston, TX, USA; 1:5000), UQCRC2 (K17L12, Selleck, Houston, TX, USA; 1:2000), MTCO2 (A18M9, Selleck, Houston, TX, USA; 1:5000), ATP5A (H18N20, Selleck, Houston, TX, USA; 1:1000), Bax (50599-2-Ig, Proteintech, Rosemont, IL, USA; 1:3000), Bcl-2 (ab12858, Abcam, Cambridge, UK; 1:2000), Caspase-9 (10380-1-AP, Proteintech, Rosemont, IL, USA; 1:1000), Cleaved caspase-9 (R381238, Zenbio, Chengdu, China; 1:1000), Caspase-3 (#14220, Cell Signaling Technology, Danvers, MA, USA; 1:1000), Cleaved caspase-3 (#9664S, Cell Signaling Technology, Danvers, MA, USA; 1:1000), Nrf2 (S2747, Selleck, Houston, TX, USA; 1:1000), and β-actin (GB15003, Servicebio, Wuhan, China; 1:3000). Original uncropped blots are provided in Supplementary File S1.

### 2.10. Quantitative Targeted Metabolomics of Energy Metabolites

H9C2 cells and rat myocardial tissues were snap-frozen and sent to Bioprofile Technology (Shanghai, China) for targeted metabolomic analysis. Metabolite extraction and LC-MS/MS analysis were performed using a QTRAP 5500 mass spectrometer (AB SCIEX, Framingham, MA, USA) coupled with a Shimadzu Nexera X2 UHPLC system (Shimadzu Corporation, Kyoto, Japan). Data were acquired in multiple reaction monitoring mode. Quality control samples were included throughout the analytical process.

### 2.11. 3D Tomographic Microscopy

H9C2 cells were seeded on 35 mm glass-bottom dishes and allowed to adhere overnight. After treatment, live cells were imaged using a 3D tomographic microscope (3D Cell Explorer, Nanolive SA, Tolochenaz, Switzerland) equipped with a 60× objective. Z-stack images were acquired, and representative focal planes were selected for evaluation of subcellular morphology. Cytoplasmic vacuolization is indicated by arrows in representative images.

### 2.12. Apoptosis Detection

H9C2 cells were seeded in 6-well plates and treated as indicated for 48 h. Apoptosis was evaluated using an Annexin V-FITC Apoptosis Detection Kit (Invitrogen, Carlsbad, CA, USA, 88-8005-72) following the manufacturer’s protocol. Cells were analyzed immediately by flow cytometry.

### 2.13. Animal Model and Treatment

Male Sprague-Dawley rats (6–8 weeks old, 200 ± 20 g) were obtained from Lanzhou Veterinary Research Institute and housed under specific pathogen-free conditions. After one week of acclimatization, rats were randomly assigned into three groups (*n* = 6 per group): Sham, CLP, and CLP + MET. Sepsis was induced by CLP according to an established protocol [25]. Briefly, rats were anesthetized with pentobarbital sodium (50 mg/kg, intraperitoneally), and the cecum was exposed through a midline laparotomy. The cecum was ligated distal to the ileocecal valve and punctured twice with a 21-gauge needle. Sham-operated animals underwent laparotomy without CLP. All rats received fluid resuscitation with pre-warmed saline (50 mL/kg, subcutaneously) immediately after surgery. Metformin (100 mg/kg, intraperitoneally) was administered 1 h after CLP in the CLP + MET group, whereas the Sham and CLP groups received an equal volume of saline. At 24 h after surgery, rats were euthanized under anesthesia, and blood and cardiac tissues were collected. All procedures were approved by the Animal Welfare Committee of Lanzhou University Second Hospital (Approval No. D2024-818) on 18 September 2024.

### 2.14. Echocardiography

Transthoracic echocardiography was performed using a Vevo 3100 imaging system (VisualSonics, Toronto, ON, Canada) equipped with an MS250 linear transducer. Rats were anesthetized with 1.5% isoflurane in oxygen during imaging. M-mode images were obtained from the parasternal long-axis view to assess left ventricular function. The following parameters were measured from at least three consecutive cardiac cycles: left ventricular ejection fraction (LVEF), left ventricular fractional shortening (LVFS), and left ventricular end-diastolic diameter (LVEDD). All measurements were performed by an investigator blinded to group allocation.

### 2.15. Hematoxylin and Eosin (H&E) Staining

Myocardial tissues were fixed in 4% paraformaldehyde, embedded in paraffin, and sectioned at 5 μm thickness. Sections were deparaffinized, rehydrated, and stained with hematoxylin followed by eosin. After dehydration and mounting, images were captured under a light microscope.

### 2.16. Immunofluorescence Staining for 8-Hydroxy-2′-Deoxyguanosine (8-OHdG)

Myocardial tissue sections (5 μm) were deparaffinized, rehydrated, and subjected to antigen retrieval. After blocking, sections were incubated overnight at 4 °C with an anti-8-OHdG primary antibody (1:200, sc-66036, Santa Cruz, Dallas, TX, USA). Following incubation with a fluorescence-conjugated secondary antibody, nuclei were counterstained with 4′,6-diamidino-2-phenylindole (DAPI, D1306, Invitrogen, Carlsbad, CA, USA). Sections were mounted with antifade medium and visualized under a fluorescence microscope.

### 2.17. Terminal Deoxynucleotidyl Transferase dUTP Nick End Labeling (TUNEL) Staining

Myocardial apoptosis was assessed using a TUNEL Apoptosis Detection Kit (C1091, Beyotime, Shanghai, China). Deparaffinized myocardial sections (5 μm) were rehydrated and incubated with the TUNEL reaction mixture according to the manufacturer’s instructions. Following the 3,3′-diaminobenzidine (DAB) chromogenic reaction and hematoxylin counterstaining, sections were examined using a light microscope. The apoptotic index was determined as the proportion of TUNEL-positive cells among the total cells in five randomly selected microscopic fields from each section.

### 2.18. Immunohistochemical Staining for Cleaved Caspase-3

Myocardial sections (5 μm) were deparaffinized, rehydrated, and subjected to antigen retrieval. Endogenous peroxidase activity was blocked using 3% hydrogen peroxide, followed by overnight incubation with an anti-cleaved caspase-3 primary antibody (1:1000, #9664S, Cell Signaling Technology, Danvers, MA, USA) at 4 °C. The sections were subsequently incubated with an HRP-conjugated secondary antibody. After DAB development, hematoxylin counterstaining was performed, and images were acquired using a light microscope.

### 2.19. Quantification of Mitochondrial DNA (mtDNA) Damage Using Long-Range PCR

mtDNA was isolated from rat myocardial tissues using a commercial extraction kit (12021, Shangbao Biotech, Shanghai, China). The integrity of mtDNA was evaluated by long-range PCR according to a previously reported method [26]. A short mitochondrial fragment encoding NADH: ubiquinone oxidoreductase core subunit 1 (*mtND1*, 202 bp) and a long mtDNA fragment (15.4 kb) were amplified for analysis. Long-range PCR amplification was conducted with GoTaq^®^ Long PCR Master Mix (M4021, Promega, Madison, WI, USA), whereas amplification of the short fragment was performed using 2× Taq Master Mix (CW0690, CWBIO, Beijing, China). PCR products were resolved on 1% agarose gels, and band intensities were quantified using ImageJ software. mtDNA damage was expressed as the amplification ratio of the long fragment to *mtND1* and normalized to the control group. The following primer sequences were used: *mtND1* forward 5′-TCCTCCTAATAAGCGGCTCCTTCTC-3′, reverse 5′-AATGGTCCTGCGGCGTATTCG-3′; mtDNA forward 5′-CACGATAGCTAAGACCCAAACTGG-3′, reverse 5′-CACCACAGTTATGTTGGTCATGGG-3′.

### 2.20. Biochemical Parameter Assays

Plasma malondialdehyde (MDA) levels and lactate dehydrogenase (LDH) activity were measured using commercial assay kits (MDA, BC0025; LDH, BC0685; Solarbio, Beijing, China) following the manufacturers’ instructions. Absorbance was measured using a microplate reader.

### 2.21. Statistical Analysis

All experiments were performed at least three times independently. Data are expressed as mean ± SD. Normality and homogeneity of variances were assessed using the Shapiro–Wilk test and Levene’s test, respectively. Comparisons among multiple groups were performed using one-way analysis of variance (ANOVA) followed by Tukey’s post hoc test, whereas comparisons between two groups were performed using two-tailed Student’s *t*-test. A value of *p* < 0.05 was considered statistically significant. Western blot band intensities were quantified with ImageJ software (v1.54g), and flow cytometry data were analyzed using FlowJo software (v10.8.1). Statistical graphs were generated with OriginPro 2019b.

## 3. Results

### 3.1. LPS Induces mtROS Production via RET at Complex I in H9C2 Cells

**Figure 1:**
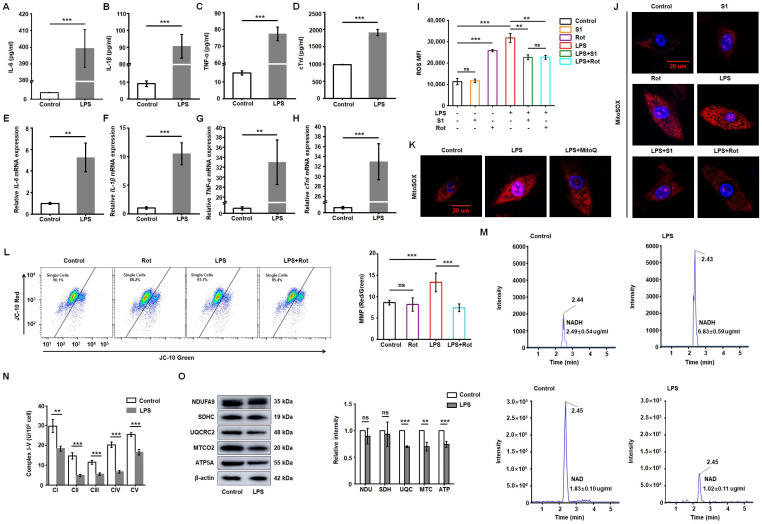
LPS induces mtROS production through complex I-mediated RET in H9C2 cells. (**A**–**D**) ELISA measurement of IL-6, IL-1β, TNF-α, and cTnI levels in culture supernatants. (**E**–**H**) Relative mRNA expression levels of *IL-6*, *IL-1β*, *TNF-α*, and *cTnI* determined by qRT-PCR. (**I**) Intracellular ROS levels detected by DCFH-DA staining and flow cytometry. (**J**,**K**) Representative confocal images of MitoSOX staining for mtROS detection in the indicated groups. Scale bar, 20 μm. (**L**) Mitochondrial membrane potential (ΔΨm) evaluated by JC-10 staining. (**M**) LC-MS/MS chromatograms of NADH and NAD^+^. (**N**) Activities of mitochondrial respiratory chain complexes I–V. (**O**) Western blot analysis of mitochondrial complex subunits (NDUFA9, SDHC, UQCRC2, MTCO2, and ATP5A). Data are presented as mean ± SD from three biological replicates. ** *p* < 0.01, *** *p* < 0.001; ns, not significant. Abbreviations: ELISA, enzyme-linked immunosorbent assay; LC-MS/MS, liquid chromatography-tandem mass spectrometry; LPS, lipopolysaccharide; MFI, mean fluorescence intensity; mtROS, mitochondrial reactive oxygen species; qRT-PCR, quantitative real-time polymerase chain reaction; RET, reverse electron transfer; Rot, rotenone; S1, S1QEL1.1.

To establish an in vitro model of SICM, H9C2 cardiomyocytes were treated with LPS (20 μg/mL). After 24 h of stimulation, secretion of the pro-inflammatory cytokines IL-6, IL-1β, and TNF-α, as well as the cardiomyocyte injury marker cTnI, was markedly enhanced (Figure 1A–D), accompanied by elevated expression of the corresponding mRNA levels (Figure 1E–H). These results confirmed successful induction of an inflammatory and injury phenotype resembling SICM.

Given the critical role of mtROS in septic organ injury, we next investigated the source of oxidative stress in LPS-treated cardiomyocytes. LPS stimulation for 2 h significantly increased intracellular ROS levels (control: 11,239 ± 1489; LPS: 31,708 ± 2168, *p* < 0.001; Figure 1I), and MitoSOX staining confirmed a mitochondrial origin of the increased ROS, with the signal fully abrogated by MitoQ treatment (Figure 1J,K). To distinguish ROS generation associated with FET from that associated with RET, we used two complementary pharmacological approaches. Rotenone, a specific inhibitor of mitochondrial complex I that interferes with electron transfer at the ubiquinone-binding site, produced opposite effects on basal and LPS-triggered ROS generation. Under basal conditions, rotenone increased ROS levels (rotenone: 25,746 ± 561, *p* < 0.001 vs. control), consistent with inhibition of FET. In contrast, rotenone significantly decreased LPS-induced ROS production (LPS + rotenone: 22,658 ± 914, *p* < 0.01 vs. LPS), which resulted from inhibition of reverse electron flow driven by the reduced CoQ pool. This opposing effect to rotenone provided initial evidence that LPS-induced ROS generation may originate from RET. To further pinpoint the ROS source, we next examined the effect of S1QEL1.1, a selective suppressor of ROS formation at the IQ site of complex I during RET that does not directly inhibit electron transport [27]. S1QEL1.1 treatment alone had no significant impact on basal ROS levels (S1QEL1.1: 11,593 ± 797, *p* = 0.735 vs. control), further supporting its lack of interference with FET. However, in LPS-stimulated cells, S1QEL1.1 substantially decreased ROS production (LPS + S1QEL1.1: 22,599 ± 1188, *p* < 0.01 vs. LPS) and attenuated MitoSOX fluorescence intensity (Figure 1I–K). Notably, the inhibitory effects of S1QEL1.1 and rotenone on LPS-induced mtROS generation were nearly identical (*p* = 0.949). These findings support a key role for complex I-mediated RET in driving LPS-induced mtROS generation in cardiomyocytes.

We further investigated whether the metabolic conditions required for RET were present following LPS stimulation. LPS treatment resulted in a significant elevation of ΔΨm (Figure 1L), providing the thermodynamic driving force for reverse electron flow toward complex I. In parallel, LPS reduced the activities of all electron transport chain (ETC) complexes and selectively decreased the protein abundance of downstream complexes III–V, whereas complexes I and II were relatively maintained (Figure 1N,O). This selective impairment of downstream electron transfer limited FET capacity and favored the accumulation of mitochondrial reducing equivalents. Consistent with this interpretation, LC-MS/MS analysis revealed a marked increase in the NADH/NAD^+^ ratio after LPS exposure (Figure 1M). This increased ratio further reflects the metabolic state associated with reverse operation of complex I, supporting the role of complex I as the site of RET-mediated mtROS generation under LPS stimulation. Collectively, these results show that LPS stimulation induces inflammatory injury and creates the conditions required for RET activation at complex I. These conditions include elevated ΔΨm, impaired downstream ETC function, and accumulation of reducing equivalents, suggesting that RET represents a major source of pathological mtROS production in this in vitro SICM model. 

### 3.2. LPS Induces Mitochondrial Metabolic Disturbances That Favor RET in H9C2 Cells

**Figure 2:**
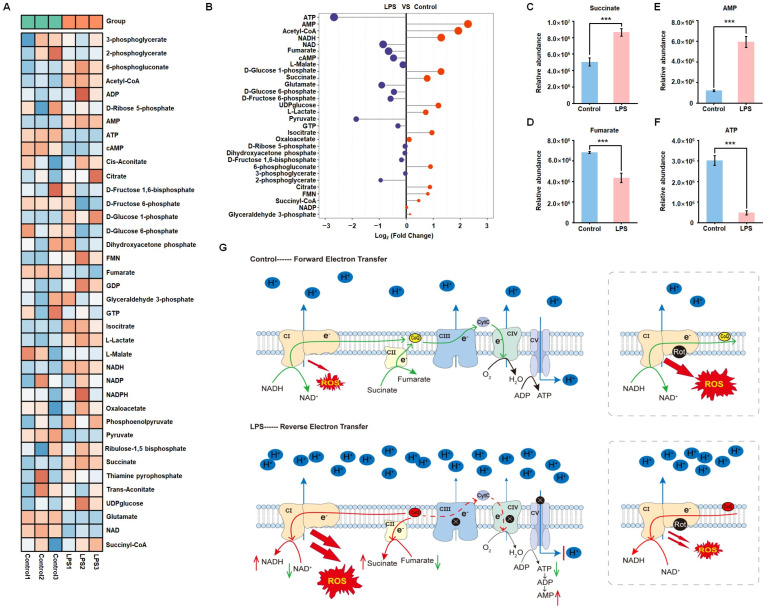
LPS induces mitochondrial metabolic remodeling associated with RET in H9C2 cells. (**A**) Hierarchical clustering heatmap of energy metabolite profiles. (**B**) VIP score analysis identifying differential metabolites between groups. (**C**–**F**) Relative levels of succinate (**C**), fumarate (**D**), AMP (**E**), and ATP (**F**). (**G**) Schematic representation of LPS-induced mitochondrial metabolic disturbances promoting reverse electron transfer at mitochondrial complex I; curved arrows indicate the direction of electron flow (red: RET; green: FET), and straight arrows indicate changes in metabolite levels (red upward: increase; green downward: decrease). Data are presented as mean ± SD from three biological replicates. *** *p* < 0.001. Abbreviations: AMP, adenosine monophosphate; ATP, adenosine triphosphate; VIP, variable importance in projection.

To investigate the metabolic changes associated with LPS-induced RET, we performed targeted energy metabolomic profiling in H9C2 cells. Hierarchical clustering demonstrated distinct metabolic patterns between control and LPS-treated cells, suggesting pronounced mitochondrial metabolic dysregulation (Figure 2A). Differential metabolite analysis based on VIP scores identified several altered metabolites (Figure 2B), among which succinate and fumarate exhibited reciprocal changes associated with RET-permissive metabolic conditions. 

Following LPS stimulation, succinate levels increased by approximately 2.16-fold, whereas fumarate levels decreased by 45.27% compared with untreated controls (Figure 2C,D). This opposite trend suggests SDH activity operating in reverse, reducing fumarate to succinate under conditions of NADH accumulation and restricted downstream electron transport. In parallel, ATP levels were substantially reduced by approximately 87.62%, accompanied by a 5.14-fold elevation in AMP levels, indicating severe impairment of mitochondrial energy homeostasis (Figure 2E,F). The increase in succinate, together with the elevated NADH/NAD^+^ ratio, may provide favorable conditions for RET at complex I. These metabolic alterations are summarized in the schematic model shown in Figure 2G. Under normal conditions, electrons were transferred sequentially through complexes I–V, supporting oxidative phosphorylation while maintaining limited mtROS production and efficient ATP synthesis. Following LPS stimulation, dysfunction of downstream respiratory complexes III–V may restrict electron transfer, whereas enhanced SDH reverse activity promotes succinate-driven electron delivery to the CoQ pool. Combined with increased ΔΨm, these alterations create a metabolic environment that facilitates complex I-mediated RET and subsequently mtROS overproduction. 

### 3.3. Metformin Reduces LPS-Induced Oxidative Stress, Inflammation, and Apoptosis by Limiting RET at Complex I in H9C2 Cells

**Figure 3:**
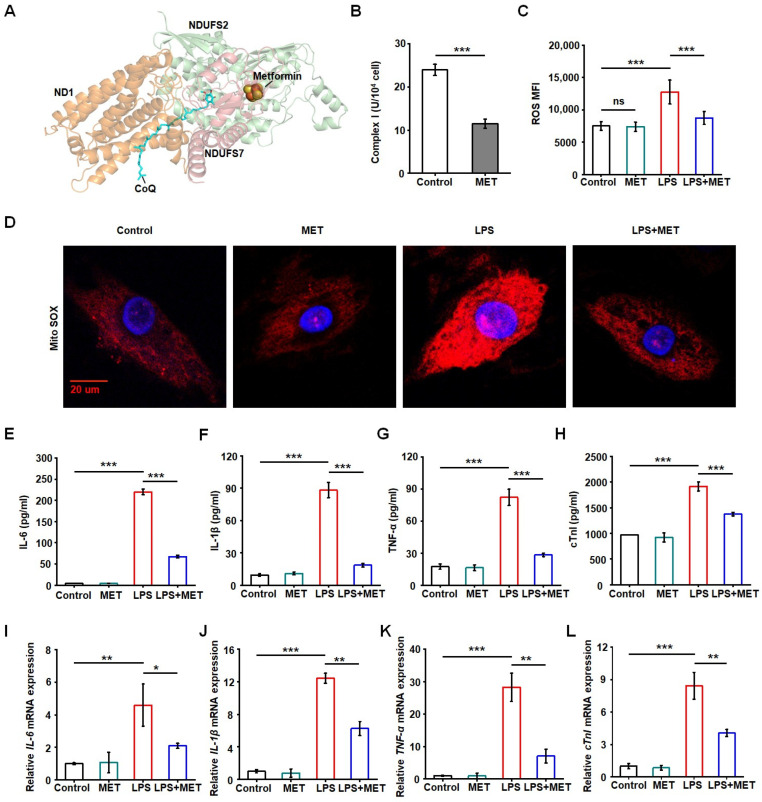
Metformin attenuates LPS-induced oxidative stress and inflammation through inhibition of complex I-mediated RET in H9C2 cells. (**A**) Structural model of metformin binding to mitochondrial complex I based on PDB 8ZSL. (**B**) Mitochondrial complex I activity in control and MET-treated cells. (**C**) Intracellular ROS levels determined by DCFH-DA staining and flow cytometry. (**D**) Representative confocal images of MitoSOX staining for mtROS detection in the indicated groups. Scale bar, 20 μm. (**E**–**H**) ELISA analysis of IL-6 (**E**), IL-1β (**F**), TNF-α (**G**), and cTnI (**H**) levels in culture supernatants. (**I**–**L**) Relative mRNA expression levels of *IL-6* (**I**), *IL-1β* (**J**), *TNF-α* (**K**), and *cTnI* (**L**). Data are presented as mean ± SD from three biological replicates. * *p* < 0.05, ** *p* < 0.01, *** *p* < 0.001. ns, not significant. Abbreviations: MET, metformin.

Metformin is a clinically well-tolerated, mild inhibitor of mitochondrial complex I that binds selectively to the open deactive conformation of the enzyme, physically obstructing electron reflux while preserving near-normal FET (Figure 3A [21]). In this study, metformin treatment modestly decreased in basal complex I activity but markedly alleviated the impairment of complex I function induced by LPS exposure (Figure 3B). Unlike rotenone, which increased ROS production, metformin alone did not increase intracellular ROS levels compared with untreated controls (control: 7499 ± 691; MET: 7380 ± 742; *p* > 0.05) (Figure 3C). However, metformin co-treatment significantly suppressed LPS-induced intracellular ROS accumulation (LPS: 12,761 ± 1847; LPS + MET: 8753 ± 1017; *p* < 0.001) and reduced MitoSOX fluorescence intensity (Figure 3C,D), supporting a reduction in RET-associated mitochondrial oxidative stress. Consistent with this effect, metformin inhibited secretion and downregulated mRNA expression of pro-inflammatory cytokines, including *IL-6*, *IL-1β*, *TNF-α* and *cTnI* in LPS-treated cardiomyocytes (Figure 3E–L). These results demonstrate that metformin protects cardiomyocytes from LPS-induced inflammatory injury through inhibition of RET-associated complex I dysfunction.

**Figure 4:**
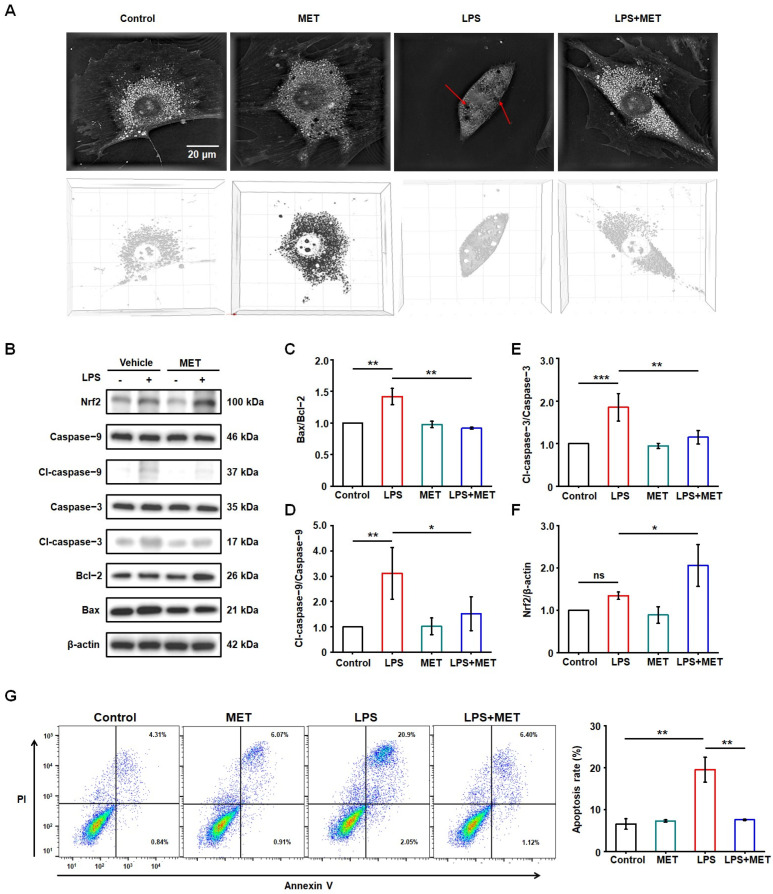
Metformin attenuates LPS-induced apoptosis in H9C2 cells. (**A**) Morphological changes in H9C2 cells visualized by 3D holotomographic microscopy; arrows indicate cytoplasmic vacuolization. Scale bar, 20 μm. (**B**) Representative Western blot bands of apoptosis-related proteins. (**C**–**F**) Quantitative analysis of Bax/Bcl-2 ratio (**C**), cleaved caspase-9/caspase-9 ratio (**D**), cleaved caspase-3/caspase-3 ratio (**E**), and Nrf2/β-actin expression level (**F**). (**G**) Apoptosis was assessed by Annexin V/PI staining followed by flow cytometry. Data are presented as mean ± SD of three biological replicates. * *p* < 0.05, ** *p* < 0.01, *** *p* < 0.001. ns, not significant.

Beyond its effects on inflammatory responses, excessive mtROS accumulation can 3also activate the intrinsic apoptotic pathway. 3D holotomographic imaging showed that LPS stimulation induced extensive cytoplasmic vacuolization and disrupted mitochondrial morphology, characterized by fragmentation and shortening of the mitochondrial network compared with the elongated tubular morphology observed in control cells. These morphological abnormalities were largely alleviated following metformin treatment (Figure 4A). Consistent with the improvement in mitochondrial morphology, metformin reduced the LPS-induced elevation of the Bax/Bcl-2 ratio and decreased the levels of cleaved caspase-9 and cleaved caspase-3 (Figure 4B–E). Metformin also increased the expression of the antioxidant regulator Nrf2 (Figure 4F). Flow cytometric analysis further confirmed that metformin significantly attenuated LPS-induced apoptosis (Figure 4G). Together, these findings suggest that metformin selectively inhibits complex I-mediated RET in LPS-treated cardiomyocytes, thereby reducing mtROS production, attenuating inflammatory responses, and preserving mitochondrial integrity and cell survival.

### 3.4. Metformin Alleviates CLP-Induced Myocardial Injury and Dysfunction Through Modulation of RET at Complex I in Rats

**Figure 5:**
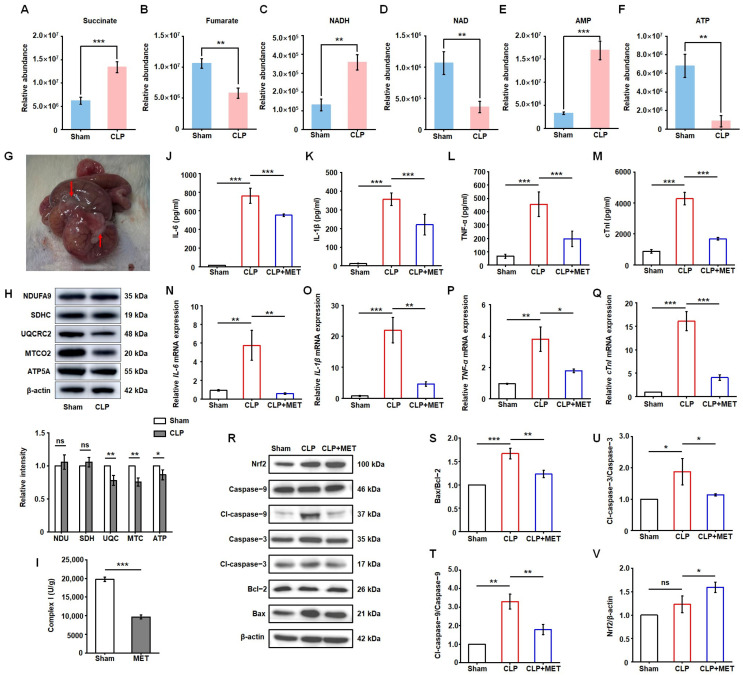
Metformin attenuates CLP-induced inflammation and apoptosis through inhibition of complex I-mediated RET in rats. (**A**–**F**) Relative levels of succinate (**A**), fumarate (**B**), NADH (**C**), NAD^+^ (**D**), AMP (**E**), and ATP (**F**) in myocardial tissues determined by LC–MS/MS (*n* = 3). (**G**) Representative photograph of CLP surgery showing purulent peritonitis (arrow). (**H**) Western blot analysis of mitochondrial complex subunits (NDUFA9, SDHC, UQCRC2, MTCO2, and ATP5A) (*n* = 3). (**I**) Mitochondrial complex I activity in sham and MET-treated rats (*n* = 3). (**J**–**M**) ELISA analysis of IL-6 (**J**), IL-1β (**K**), TNF-α (**L**), and cTnI (**M**) levels in serum (*n* = 6). (**N**–**Q**) qRT-PCR analysis of the relative mRNA expression levels of *IL-6* (**N**), *IL-1β* (**O**), *TNF-α* (**P**), and *cTnI* (Q) (*n* = 3). (**R**) Representative Western blot bands of apoptosis-related proteins (*n* = 3). (**S**–**V**) Quantitative analysis of Bax/Bcl-2 ratio (**S**), cleaved caspase-9/caspase-9 ratio (**T**), cleaved caspase-3/caspase-3 ratio (**U**), and Nrf2/β-actin expression level (**V**) (*n* = 3). Data are presented as mean ± SD. * *p* < 0.05, ** *p* < 0.01, *** *p* < 0.001. ns, not significant. Abbreviations: CLP, cecal ligation and puncture.

To determine whether RET contributes to sepsis-induced myocardial injury in vivo, a CLP-induced septic rat model was established (Figure 5G). We first examined whether the metabolic environment required for RET activation was present in septic myocardium. Targeted metabolomic analysis showed that CLP caused a significant increase in myocardial succinate levels, a reduction in fumarate levels, and an increased NADH/NAD^+^ ratio (Figure 5A–D), consistent with the metabolic alterations observed in vitro. In addition, septic myocardial tissue exhibited reduced ATP production, increased AMP accumulation, and selective downregulation of ETC complexes III–V (Figure 5E,F,H). These findings indicate that sepsis induces metabolic and bioenergetic changes that favor RET activation in the myocardium.

Next, we examined the effects of metformin treatment in this model. Metformin suppressed myocardial complex I activity (Figure 5I) and alleviated CLP-induced increases in serum and myocardial pro-inflammatory cytokines and cTnI levels (Figure 5J–Q). Moreover, metformin reduced the Bax/Bcl-2 ratio, decreased caspase-9 and caspase-3 cleavage, and enhanced Nrf2 expression in septic myocardium (Figure 5R–V), supporting its protective effects against apoptosis and oxidative stress observed in vitro.

**Figure 6:**
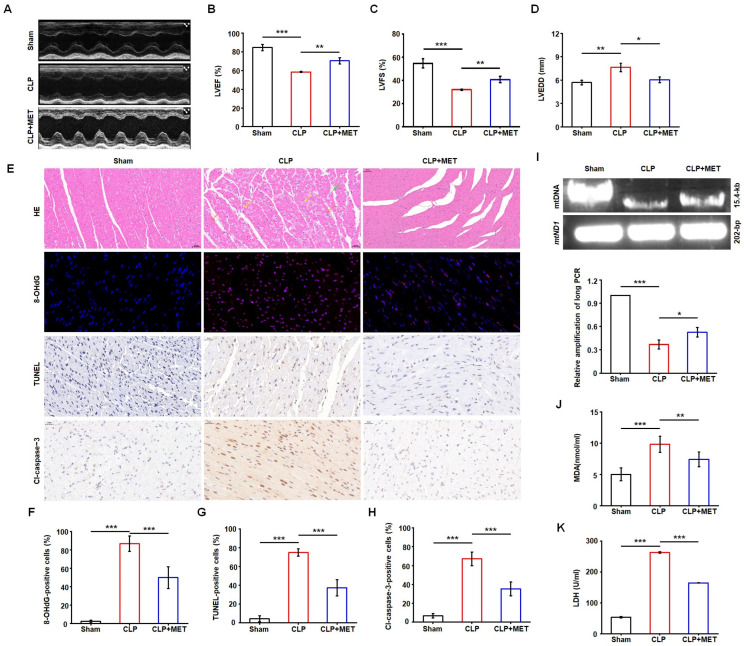
Metformin protects against CLP-induced myocardial injury and dysfunction in rats. (**A**) Representative M-mode echocardiographic images (*n* = 6). (**B**–**D**) Quantitative analysis of LVEF (**B**), LVFS (**C**), and LVEDD (**D**) (*n* = 6). (**E**) Representative images of myocardial sections subjected to H&E, 8-OHdG, TUNEL, and cleaved caspase-3 staining. Blue fluorescence indicates nuclei (DAPI), red fluorescence indicates 8-OHdG-positive signals, and brown staining indicates positive cells for TUNEL and cleaved caspase-3. In the H&E staining panels, yellow arrows indicate myocardial edema, orange arrows indicate myocardial necrosis, and green arrows indicate lymphocyte infiltration. Scale bars: 50 μm for H&E staining and 20 μm for other staining images (*n* = 3). (**F**–**H**) Quantitative analysis of 8-OHdG-positive cells (**F**), TUNEL-positive cells (**G**), and cleaved caspase-3-positive cells (**H**) (*n* = 3). (**I**) Long-range PCR analysis of mitochondrial DNA damage (*n* = 3). (**J**,**K**) Plasma MDA levels (**J**) and LDH activity (**K**) determined using commercial assay kits (*n* = 6). Data are presented as mean ± SD. * *p* < 0.05, ** *p* < 0.01, *** *p* < 0.001. Abbreviations: 8-OHdG, 8-hydroxy-2′-deoxyguanosine; DAPI, 4′,6-diamidino-2-phenylindole; H&E, hematoxylin and eosin; LDH, lactate dehydrogenase; LVEDD, left ventricular end-diastolic diameter; LVEF, left ventricular ejection fraction; LVFS, left ventricular fractional shortening; MDA, malondialdehyde; TUNEL, terminal deoxynucleotidyl transferase dUTP nick end labeling.

We further examined whether metformin-mediated metabolic protection was associated with improved myocardial structure and function. Echocardiographic analysis demonstrated that metformin preserved left ventricular systolic function in CLP-treated rats, as evidenced by increased left ventricular ejection fraction (LVEF) and fractional shortening (LVFS), and attenuated left ventricular end-diastolic diameter (LVEDD) enlargement (Figure 6A–D). Histological examination showed that metformin maintained myocardial architecture, reduced oxidative DNA damage as indicated by 8-OHdG staining, and reduced apoptosis, as demonstrated by TUNEL staining and cleaved caspase-3 immunoreactivity (Figure 6E–H). Long-range PCR analysis further showed that metformin attenuated CLP-induced mtDNA damage (Figure 6I). Similarly, metformin reduced plasma MDA levels and LDH activity (Figure 6J,K), suggesting attenuated lipid peroxidation and cellular injury. Together, these findings suggest that septic myocardium undergoes metabolic remodeling consistent with RET activation, similar to that observed in vitro. In addition, metformin-mediated reductions in inflammation and apoptosis, along with preserved cardiac function, are in line with RET inhibition as a contributing mechanism. Collectively, these data suggest that RET may represent a therapeutic target for SICM.

## 4. Discussion

In this study, we provide evidence suggesting that RET at mitochondrial complex I contributes to SICM and support metformin as a clinically available strategy for targeting this pathway. By integrating complementary in vitro and in vivo sepsis models, we demonstrate that sepsis induces metabolic reprogramming in the myocardium, characterized by succinate accumulation, an increased NADH/NAD^+^ ratio, and selective impairment of downstream ETC complexes. This metabolic remodeling establishes conditions favorable for RET at complex I, resulting in excessive mtROS production, myocardial inflammation, apoptosis, and subsequent cardiac dysfunction. Importantly, our findings show that metformin selectively limits RET at complex I without disrupting physiological FET, thereby alleviating these pathological changes and maintaining cardiac contractile function in septic rats. Collectively, these findings provide new mechanistic insights into SICM pathogenesis and support further investigation of metformin as a therapeutic approach for sepsis-associated cardiac injury.

The metabolic alterations identified here are consistent with the major requirements for RET activation at complex I, namely a high proton motive force and an abundant supply of reducing equivalents that facilitate reverse electron flow [28]. In LPS-stimulated H9C2 cells, we observed a pronounced increase in ΔΨm (Figure 1L), which provides the energetic driving force necessary for electron transfer reversal toward complex I [29]. Moreover, metabolomic analysis revealed substantial succinate accumulation accompanied by reciprocal fumarate depletion (Figure 2C,D and Figure 5A,B). This metabolic pattern is consistent with reverse SDH activity, which may promote reduction in the CoQ pool, a key intermediate involved in RET [30]. Further evidence supporting the formation of RET-permissive conditions included a high NADH/NAD^+^ ratio (Figure 1M and Figure 5C,D) and selective downregulation of ETC complexes III–V (Figure 1O and Figure 5H). These changes may restrict FET and promote a highly reduced mitochondrial matrix state, thereby facilitating RET occurrence [31,32]. Pharmacological experiments using inhibitors with distinct selectivity profiles further support this interpretation. The opposite effects of rotenone under basal and LPS-stimulated conditions, together with the selective reduction in LPS-induced mtROS by S1QEL1.1 and its comparable effect to rotenone, are consistent with RET as a major contributor to LPS-induced mtROS production. Although this RET-permissive metabolic profile resembles that reported in ischemia–reperfusion injury [8], our findings further reveal a more severe bioenergetic impairment in septic myocardium [33], as demonstrated by substantial ATP depletion and AMP accumulation (Figure 2E,F and Figure 5E,F). These results expand the current understanding of SICM pathogenesis by highlighting mitochondrial metabolic failure as an additional pathogenic axis beyond immune dysregulation, which has traditionally been regarded as a central driver of sepsis-associated organ injury [34]. Our findings suggest that SICM involves a mitochondrial energy disorder characterized by impaired electron transfer and disrupted metabolic homeostasis. The metabolic characteristics identified may help identify septic patients who are more likely to benefit from RET-targeted interventions, including metformin, and support a more individualized therapeutic strategy.

The therapeutic effects of metformin in SICM may be attributed to its unique pattern of complex I inhibition, which differs from that of traditional complex I inhibitors such as rotenone. Unlike rotenone, which binds to the ubiquinone site of complex I in both active and deactive states and inhibits FET, metformin preferentially interacts with complex I in the open deactive conformation and becomes trapped at the ubiquinone redox site following substrate-induced closure [21,35]. This interaction pattern allows metformin to limit electron reflux from the reduced CoQ pool while maintaining relatively normal physiological respiration. Consistent with this mechanism, our results showed that metformin caused only a moderate reduction in basal complex I activity (Figure 3B and Figure 5I), but effectively inhibited LPS-induced mtROS production without increasing basal ROS levels (Figure 3C,D). Beyond its mitochondrial effects, metformin has been widely used in clinical practice, with established pharmacological characteristics and safety profiles, supporting its potential application in SICM. However, several issues require further consideration before clinical translation. In particular, the dose required to achieve effective RET inhibition in septic patients remains unclear. Moreover, impaired hepatic or renal function during sepsis and the potential risk of lactic acidosis may affect metformin metabolism, tolerance, and safety in this critically ill population.

The inhibitory effect of metformin on RET may be enhanced by the metabolic environment in the septic myocardium, resulting in a “pathology-selective” therapeutic effect. Under normal physiological states, complex I mainly exists in its active state and the CoQ pool remains relatively oxidized. In this condition, metformin acts as a weak and non-competitive inhibitor because the catalytic transition of complex I allows the inhibitor to dissociate when the enzyme returns to the open state [36]. In contrast, during septic stress, complex I is more likely to accumulate in the open deactive conformation, accompanied by a high reduction in the CoQ pool. These changes may facilitate metformin binding to complex I and enhance its inhibitory effect through interactions with the reduced CoQ pool [36]. Furthermore, the high ΔΨm observed in septic cardiomyocytes may promote the accumulation of positively charged metformin within the mitochondrial matrix, thereby increasing its local availability [37]. This metabolic environment may explain how a mild complex I inhibitor can exert substantial cardioprotective effects during sepsis. From a therapeutic perspective, this potential pathology-selective effect is highly desirable, as it suggests that metformin may preferentially act under pathological conditions while limiting effects on healthy myocardial tissue and reducing potential off-target effects.

Beyond its direct effects on RET, metformin may exert cardioprotective effects through several other mitochondrial and cellular mechanisms [9]. Cameron et al. reported that metformin induces a functional uncoupling state between the redox and proton transfer domains of complex I, thereby promoting oxidation of the mitochondrial NADH/NAD^+^ couple without causing mitochondrial injury [38]. In our study, sepsis-induced elevation of the NADH/NAD^+^ ratio generated a reduced mitochondrial matrix environment that favors RET energetically [39]. Therefore, metformin-mediated oxidation of this reducing pool may decrease the driving force for reverse electron flow [14]. Furthermore, moderate inhibition of complex I by metformin may reduce excessive ATP consumption caused by RET-associated proton cycling while maintaining sufficient forward respiratory activity to support cellular energy balance [40]. Metformin has also been reported to activate AMPK, regulate autophagy, and exert anti-inflammatory effects [41,42,43]. However, these effects may be partially secondary to reduced mtROS production rather than representing completely independent mechanisms [44,45]. These pathways may function together with RET inhibition in a coordinated manner to contribute to the protective effects observed in our models. Nevertheless, our findings suggest that RET inhibition represents an important component of metformin-mediated cardioprotection. This mechanism differs from nonspecific antioxidant strategies that mainly remove ROS without addressing the underlying metabolic disturbances, which have shown limited success in improving sepsis outcomes [46]. Therefore, these findings further support RET as a potential therapeutic target for SICM.

Several limitations of the present study should be acknowledged. First, the CoQH_2_/CoQ ratio, which directly indicates the redox state of the electron donor involved in RET, was not measured in this study. However, the combined findings of succinate accumulation, increased NADH/NAD^+^ ratio, and impaired downstream ETC complexes indicate a metabolic state consistent with a reduced CoQ pool. Direct measurement of CoQ redox status in future studies would provide further evidence to support this proposed mechanism. Second, although metformin was used as a pharmacological inhibitor of complex I, genetic approaches to targeting complex I subunits were not performed. Given the reported preference of metformin for the deactive conformation of complex I and the consistency between our in vivo and in vitro findings, our results support a role for RET inhibition in metformin-mediated cardioprotection. However, future studies using cardiomyocyte-specific deletion of complex I subunits, such as Ndufs2, will be necessary to further confirm the causal relationship between RET suppression and the protective effects of metformin. Third, the in vitro experiments were performed using H9C2 cells, a cardiomyoblast-derived cell line that does not fully reproduce the mitochondrial metabolic characteristics and contractile properties of adult cardiomyocytes. Although H9C2 cells are widely used in studies of cardiomyocyte injury, validation using more physiological models, such as primary adult cardiomyocytes, will be important to further confirm our findings. Fourth, survival outcomes were not evaluated in this study. Although metformin improved cardiac function and alleviated myocardial injury at 24 h after CLP, whether these effects can ultimately improve survival during sepsis remains unknown and requires further investigation. Fifth, the in vivo experiments were performed using a single metformin dose (100 mg/kg) based on previous studies, and treatment was initiated prophylactically at 1 h after CLP. This experimental design does not fully represent clinical conditions, as septic patients usually receive treatment after disease onset and exhibit variable degrees of organ dysfunction. Therefore, whether delayed administration or dose optimization can provide similar protective effects remains to be determined. Establishing the dose–response relationship and therapeutic window will be essential for future clinical translation. Moreover, the current study was limited to young male rats and a single observation time point of 24 h. Further studies are needed to determine whether the protective effects of metformin are maintained across different sexes and during the later stages of sepsis. Addressing these limitations will be important for evaluating the clinical potential of metformin-based therapeutic strategies for SICM.

## 5. Conclusions

In summary, this study suggests that RET at mitochondrial complex I acts as a pathogenic mechanism contributing to SICM and that metformin protects against septic myocardial injury, in part through conformation-dependent inhibition of RET. The excellent safety profile, pathology-selective activity, and complementary metabolic effects of metformin position it as a promising candidate for clinical translation. Given the lack of effective targeted therapies for SICM, our findings provide a preclinical basis for future studies evaluating the efficacy of metformin in patients with sepsis-associated cardiac dysfunction.

## Data Availability

The data generated in this study are available within the article.

## Supplementary Materials

The following supporting information can be downloaded at: https://www.mdpi.com/article/10.3390/biology15151267/s1. Supplementary File S1: The original uncropped Western blots.

## Abbreviations

The following abbreviations are used in this manuscript:

8-OHdG	8-hydroxy-2′-deoxyguanosine
AMP	adenosine monophosphate
ANOVA	analysis of variance
ATP	adenosine triphosphate
CLP	cecal ligation and puncture
CoQ	coenzyme Q
DAB	3,3′-diaminobenzidine
DAPI	4′,6-diamidino-2-phenylindole
DCFH-DA	2′,7′-dichlorodihydrofluorescein diacetate
DMEM	Dulbecco’s Modified Eagle Medium
ELISA	enzyme-linked immunosorbent assay
ETC	electron transport chain
FET	forward electron transfer
H&E	hematoxylin and eosin
HRP	horseradish peroxidase
LDH	lactate dehydrogenase
LC–MS/MS	liquid chromatography–tandem mass spectrometry
LPS	lipopolysaccharide
LVEDD	left ventricular end-diastolic diameter
LVEF	left ventricular ejection fraction
LVFS	left ventricular fractional shortening
MDA	malondialdehyde
MET	metformin
MitoSOX	mitochondrial superoxide indicator
mtDNA	mitochondrial DNA
mtND1	mitochondrially encoded NADH:ubiquinone oxidoreductase core subunit 1
mtROS	mitochondrial reactive oxygen species
PBS	phosphate-buffered saline
qRT-PCR	quantitative real-time polymerase chain reaction
RET	reverse electron transfer
ROS	reactive oxygen species
Rot	rotenone
SDH	succinate dehydrogenase
SDS-PAGE	sodium dodecyl sulfate-polyacrylamide gel electrophoresis
SICM	sepsis-induced cardiomyopathy
TUNEL	terminal deoxynucleotidyl transferase dUTP nick end labeling
VIP	variable importance in projection
ΔΨm	mitochondrial membrane potential
