# Supplementary material for: Metformin Attenuates Sepsis-Induced Cardiomyopathy via Inhibition of Reverse Electron Transfer at Mitochondrial Complex I
Source: Biology (Basel). 2026 Aug 2;15(15):1267. doi: 10.3390/biology15151267 (PMC13463947; doi:10.3390/biology15151267)

Figure 1O

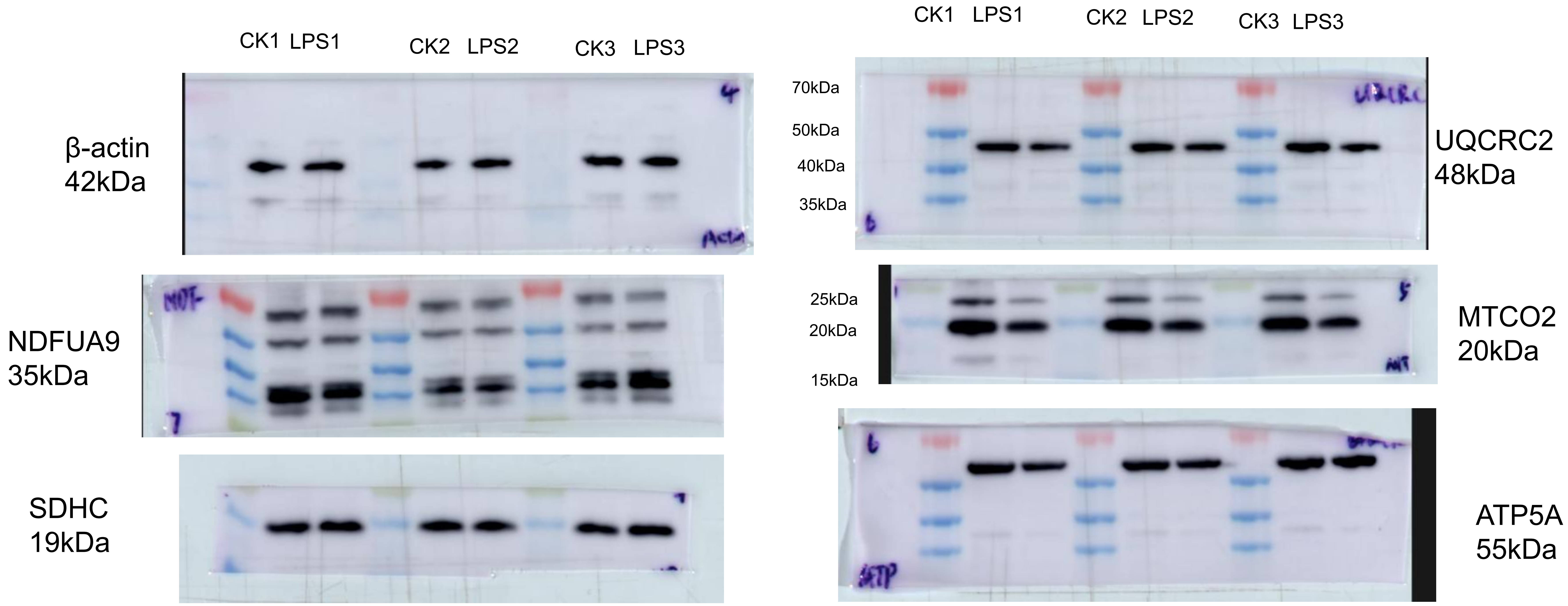
CK1 LPS1 CK2 LPS2 CK3 LPS3
β-actin
42kDa
NDFUA9
35kDa
SDHC
19kDa
CK1 LPS1 CK2 LPS2 CK3 LPS3
70kDa
50kDa
40kDa
35kDa
UQCRC2
48kDa
25kDa
20kDa
15kDa
MTCO2
20kDa
ATP5A
55kDa

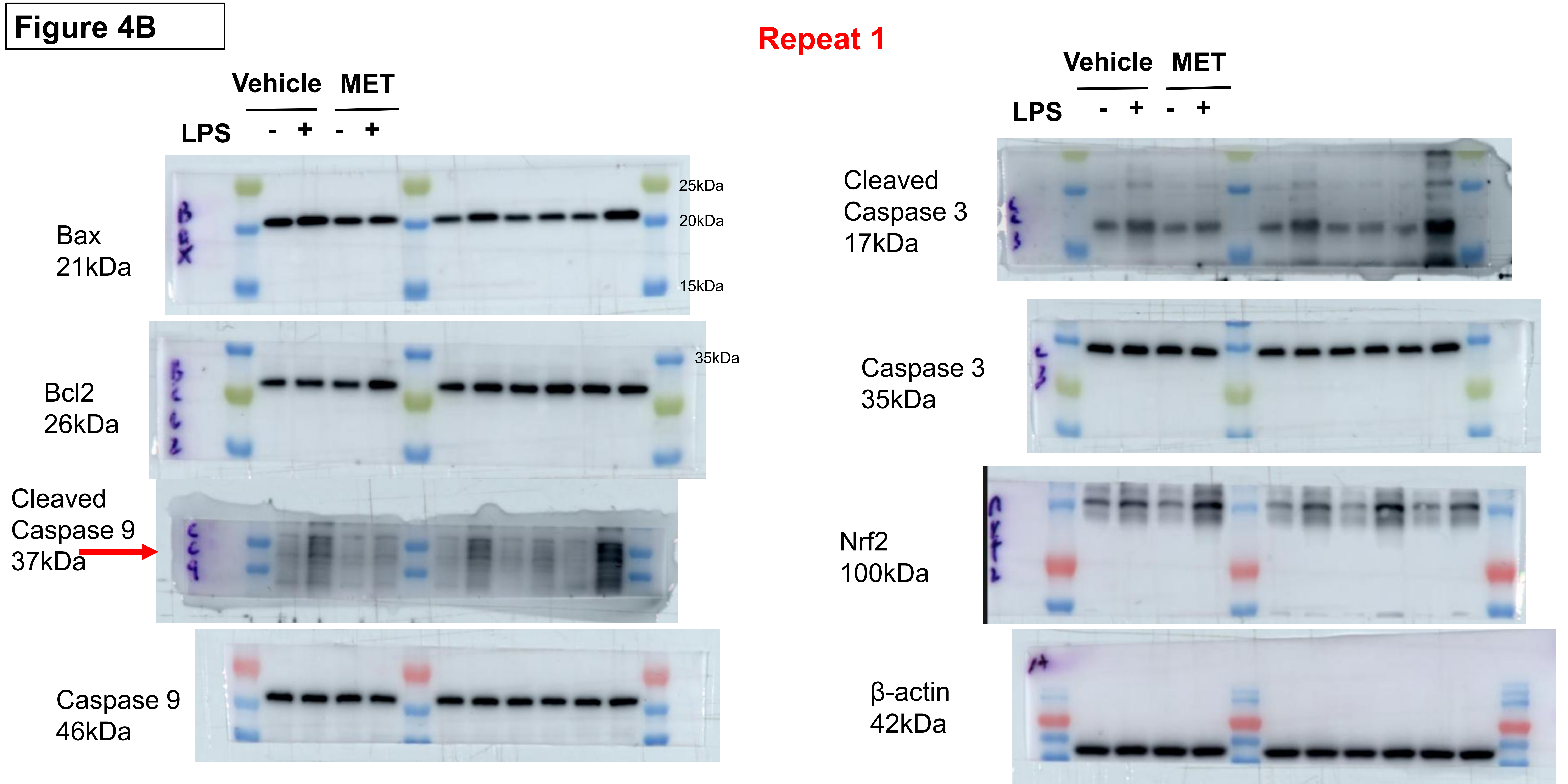

Figure 4B
Repeat 1
Vehicle
MET
LPS
- + - +
Bax
21kDa
25kDa
20kDa
15kDa
Bcl2
26kDa
35kDa
Cleaved
Caspase 9
37kDa
Caspase 9
46kDa
Vehicle
MET
LPS
- + - +
Cleaved
Caspase 3
17kDa
Caspase 3
35kDa
Nrf2
100kDa
β-actin
42kDa

Figure 4B

Repeat 2

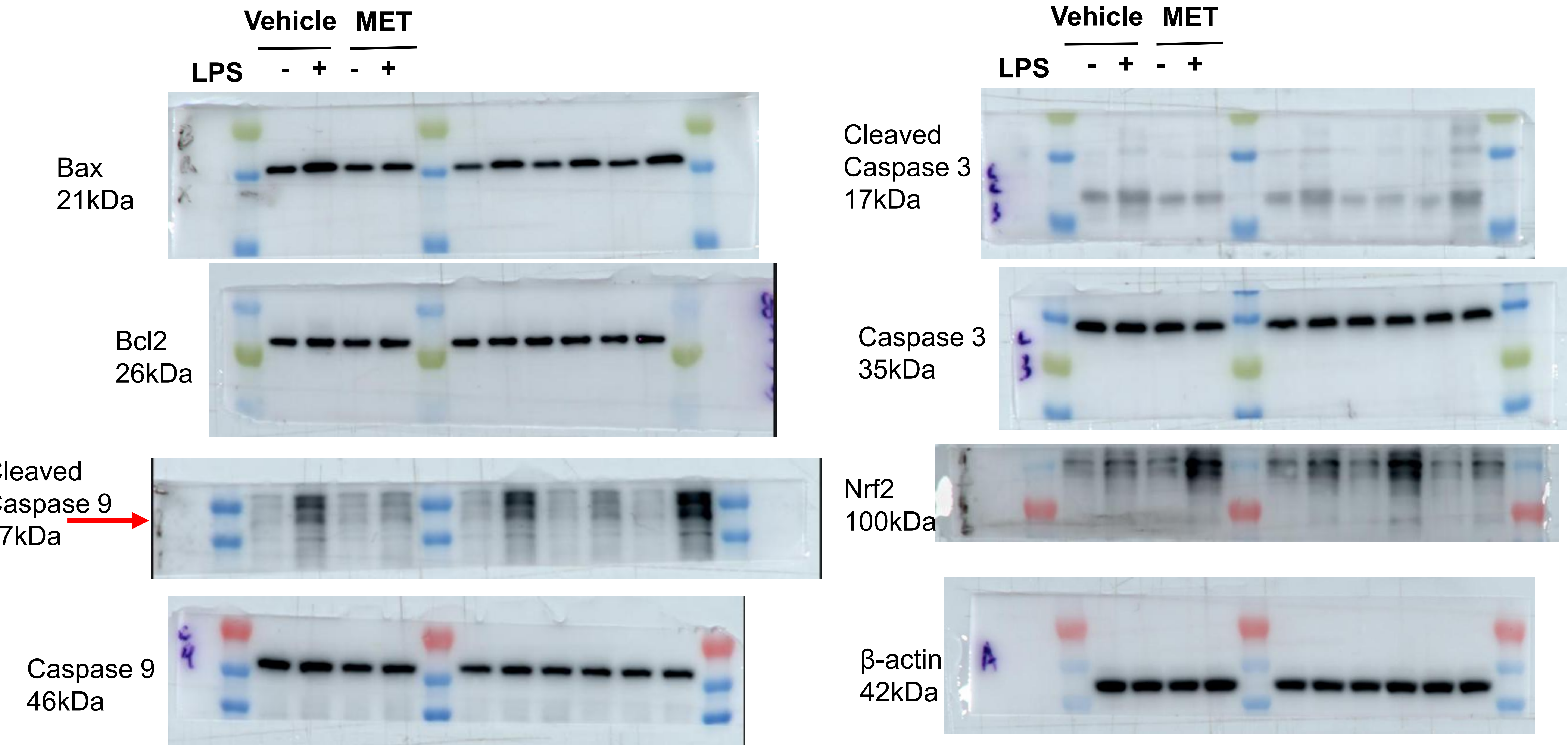

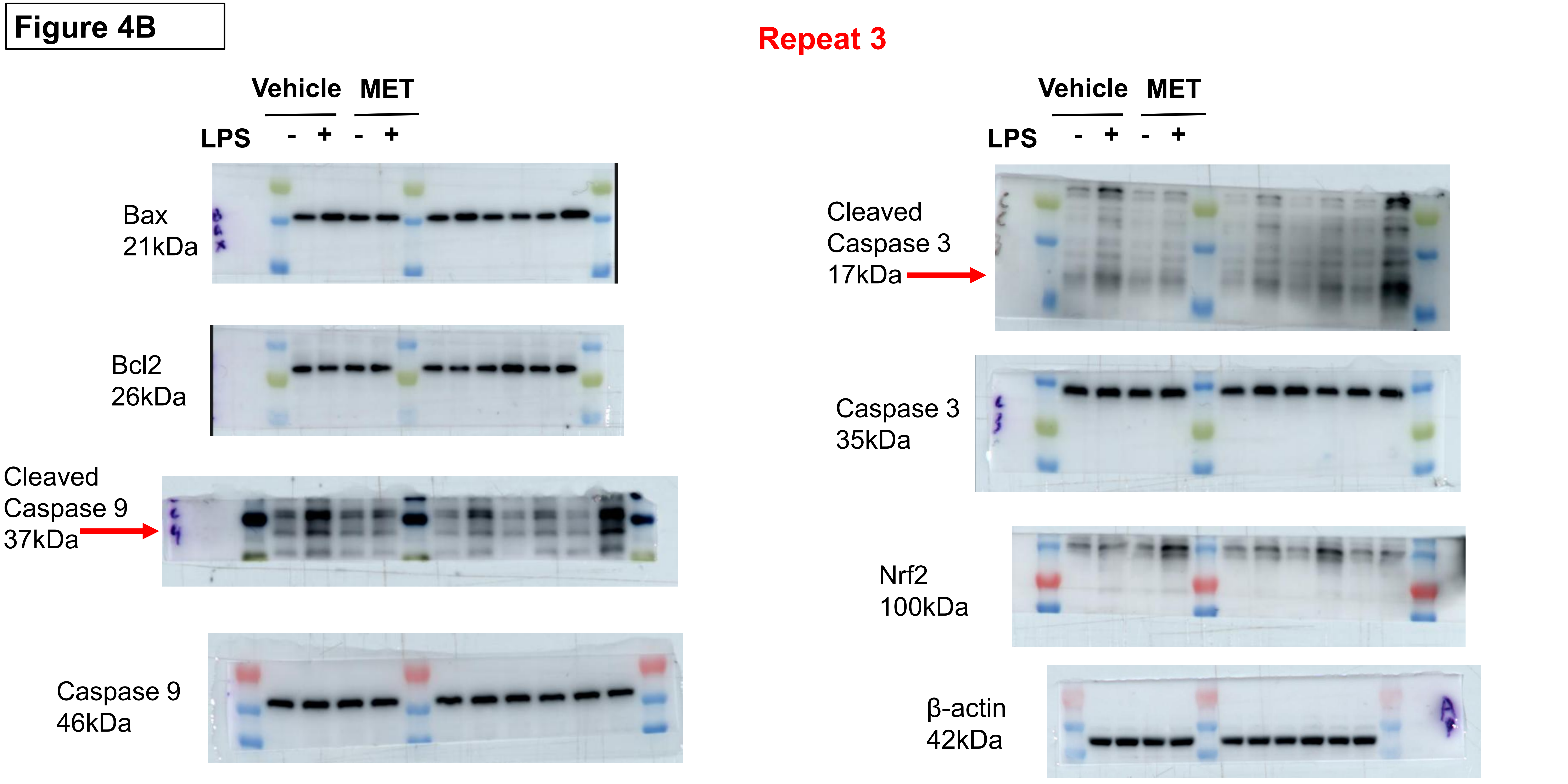

Figure 4B
Repeat 3
Vehicle
MET
LPS - + - +
Bax
21kDa
Bcl2
26kDa
Cleaved
Caspase 9
37kDa
Caspase 9
46kDa
Vehicle
MET
LPS - + - +
Cleaved
Caspase 3
17kDa
Caspase 3
35kDa
Nrf2
100kDa
β-actin
42kDa

Figure 5H

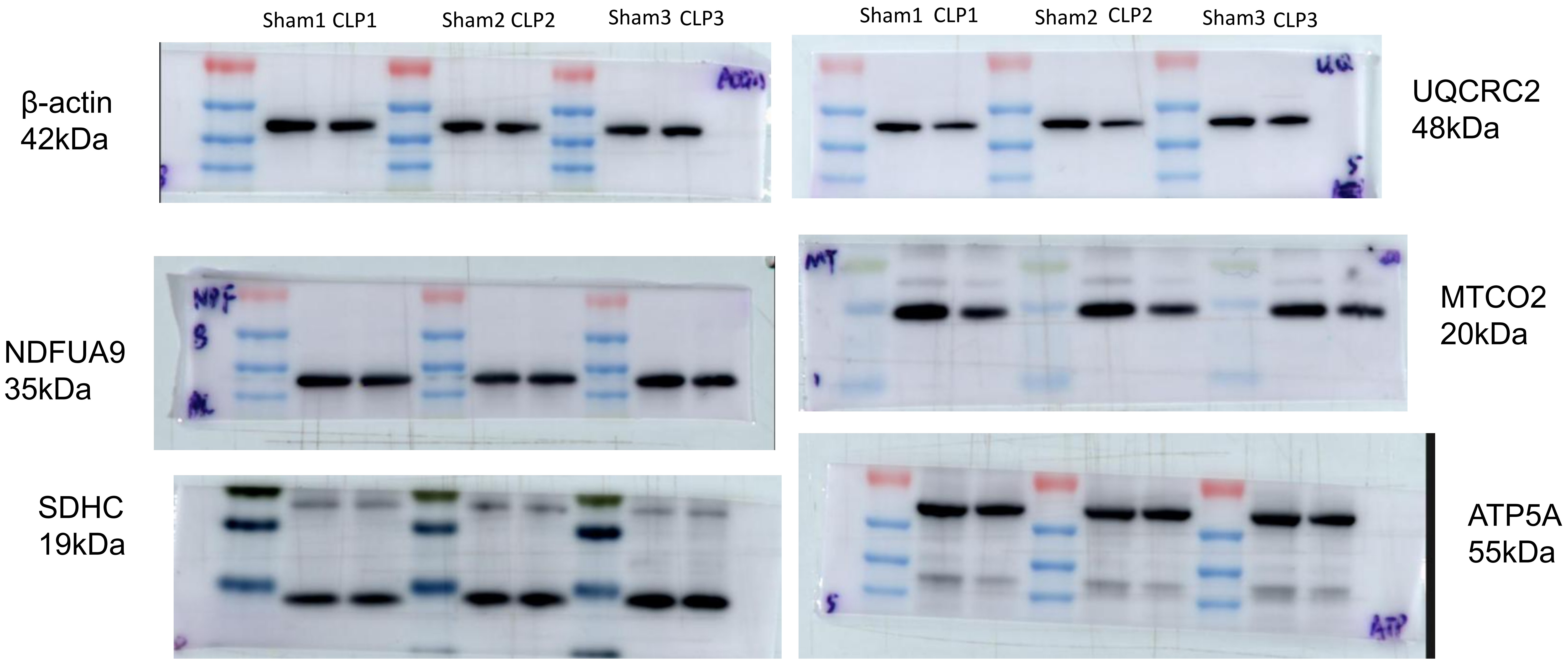
Sham1 CLP1 Sham2 CLP2 Sham3 CLP3
Sham1 CLP1 Sham2 CLP2 Sham3 CLP3
β-actin
42kDa
UQCRC2
48kDa
NDFUA9
35kDa
MTCO2
20kDa
SDHC
19kDa
ATP5A
55kDa

Figure 5R

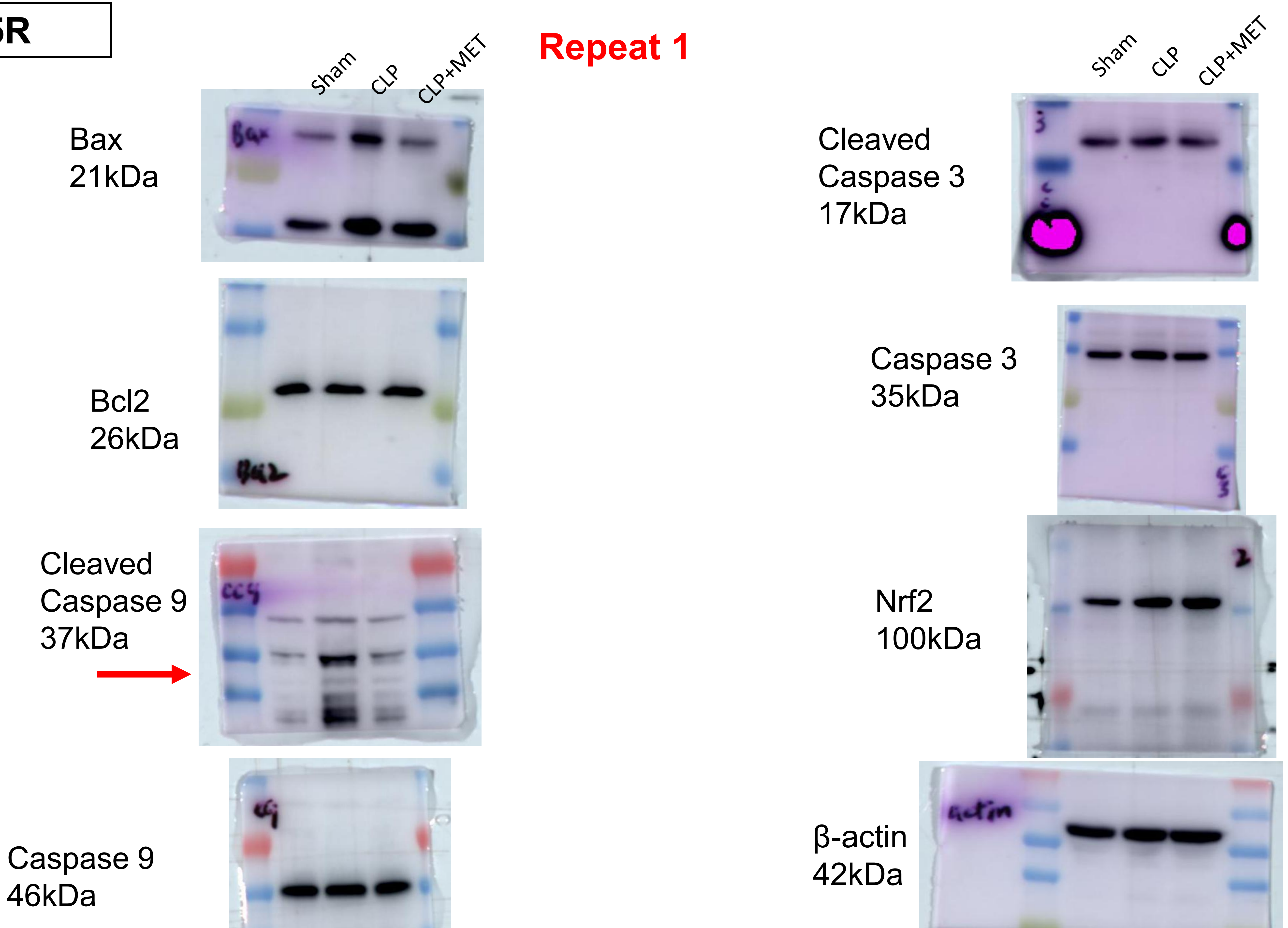
Repeat 1
Sham
CLP
CLP+MET
Bax
21kDa
Bcl2
26kDa
Cleaved
Caspase 9
37kDa
Caspase 9
46kDa
Sham
CLP
CLP+MET
Cleaved
Caspase 3
17kDa
Caspase 3
35kDa
Nrf2
100kDa
β-actin
42kDa

Figure 5R

Repeat 2

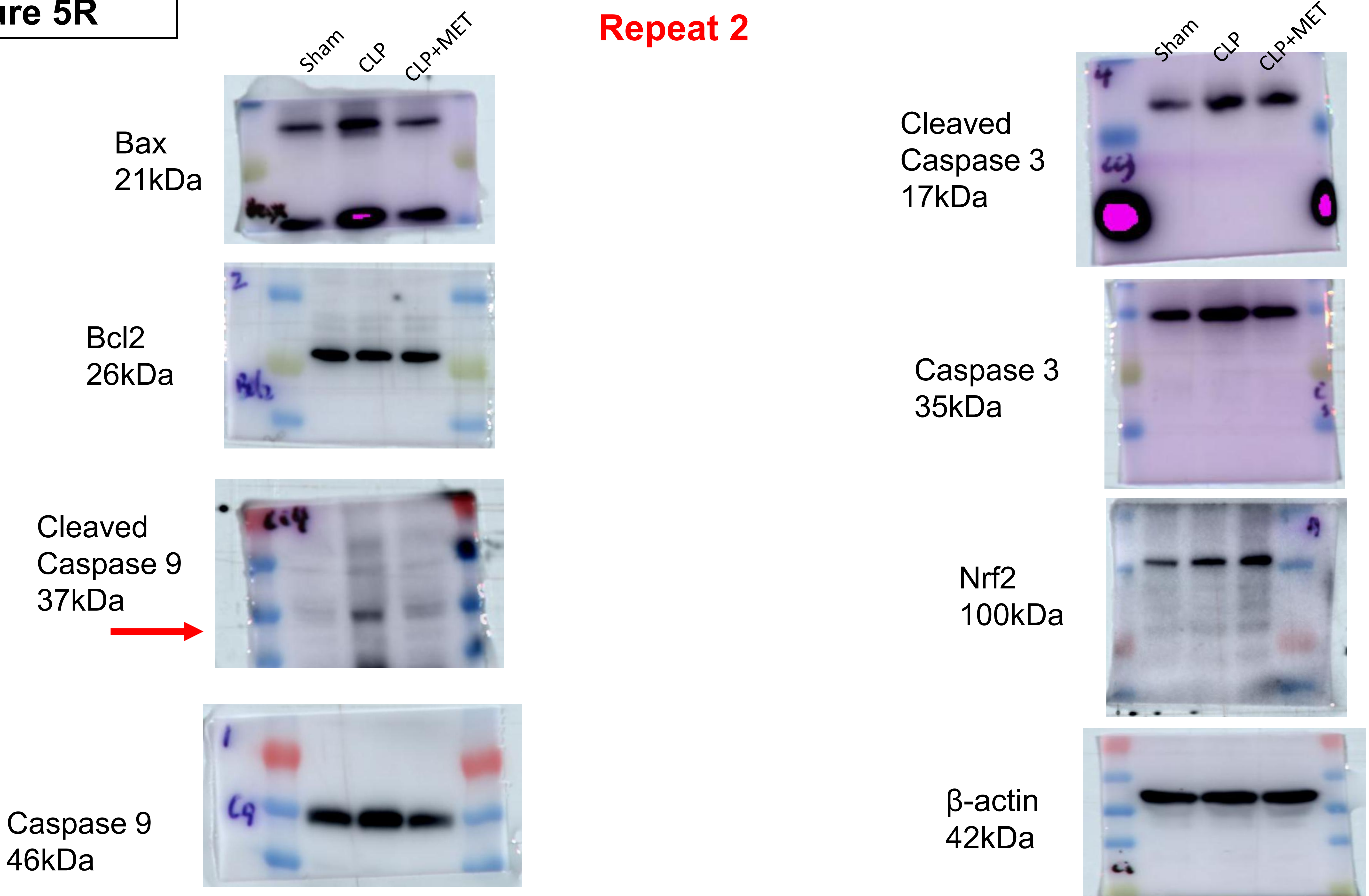

Figure 5R

Repeat 3

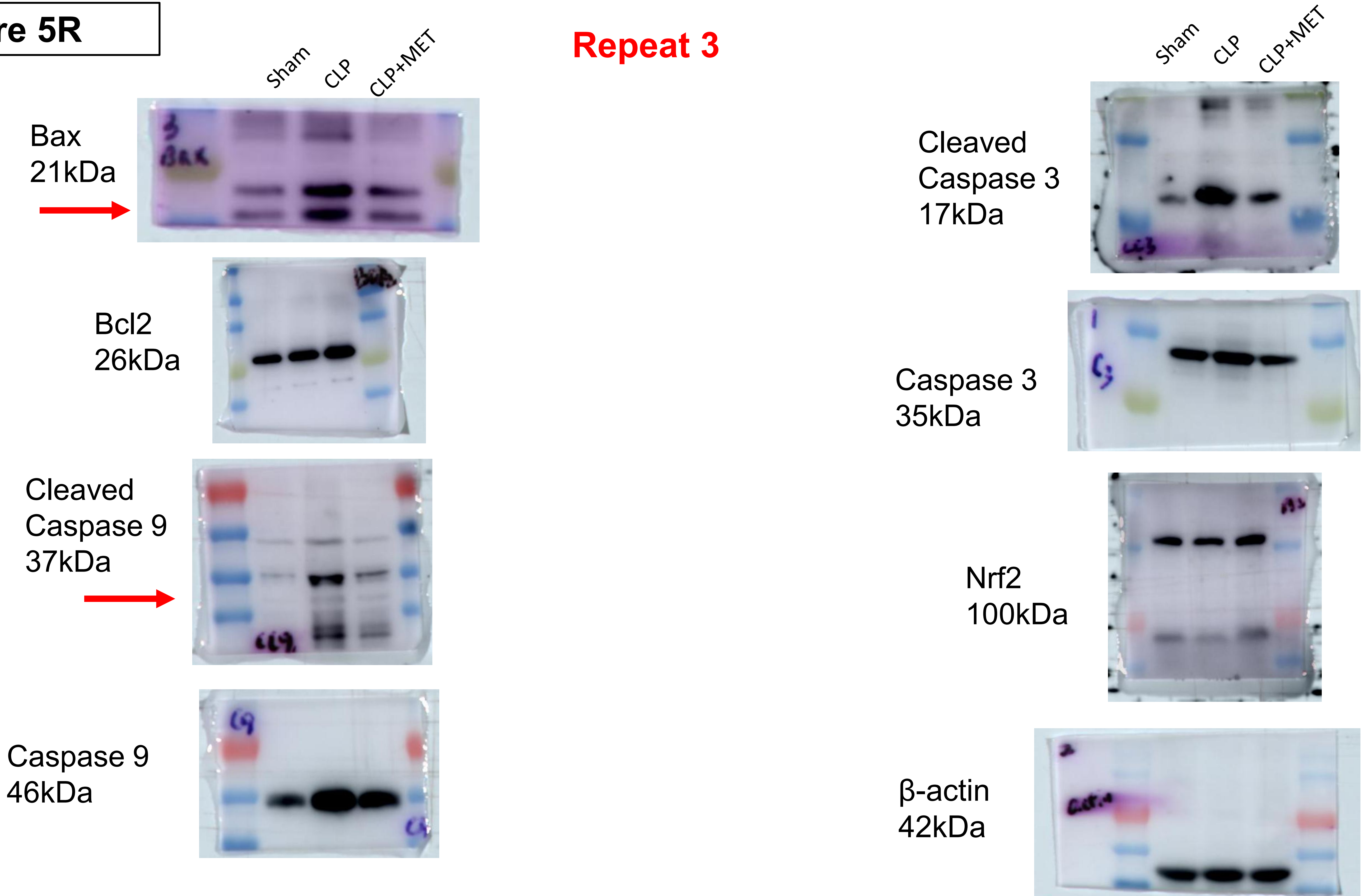

Supplement: Supplementary file 1 [file biology-15-01267-s001.zip › biology-4359622-supplementary.pdf]
